# The Bromodomain Inhibitor, INCB057643, Targets Both Cancer Cells and the Tumor Microenvironment in Two Preclinical Models of Pancreatic Cancer

**DOI:** 10.3390/cancers13010096

**Published:** 2020-12-30

**Authors:** Ana S. Leal, Phillip Liu, Teresa Krieger-Burke, Bruce Ruggeri, Karen T. Liby

**Affiliations:** 1 Department of Pharmacology & Toxicology, Michigan State University, B430 Life Science Building, 1355 Bogue Street, East Lansing, MI 48824, USA; mendesle@msu.edu (A.S.L.); kriege29@msu.edu (T.K.-B.); 2 Incyte Corporation, Wilmington, DE 19803, USA; pliu@kymeratx.com (P.L.); bruggeri@preludetx.com (B.R.)

**Keywords:** pancreatic cancer, bromodomain inhibitors, inflammatory tumor microenvironment

## Abstract

**Simple Summary:**

Pancreatic cancer remains a highly lethal disease, with only ~10% of patients still alive five years after diagnosis, as most patients already have advanced, metastatic disease at the time of diagnosis. Therefore, new treatments are needed for these patients. We tested INCB057643, a novel bromodomain inhibitor, in a relevant mouse model of pancreatic cancer, and this compound improves survival and reduces metastasis. Pancreatic cancers are very dense, as the stroma within the tumor can account for up to 90% of the tumor mass and is responsible for the failure of many drugs. INCB057643 modulates the immune cells within the tumor so they can attack and kill tumor cells. INCB057643 also alters immune cells within the pancreas in a mouse model of pancreatitis, which is inflammation of the pancreas that can promote the development of pancreatic cancer.

**Abstract:**

In pancreatic cancer the tumor microenvironment (TME) can account for up to 90% of the tumor mass. The TME drives essential functions in disease progression, invasion and metastasis. Tumor cells can use epigenetic modulation to evade immune recognition and shape the TME toward an immunosuppressive phenotype. Bromodomain inhibitors are a class of drugs that target BET (bromodomain and extra-terminal) proteins, impairing their ability to bind to acetylated lysines and therefore interfering with transcriptional initiation and elongation. INCB057643 is a new generation, orally bioavailable BET inhibitor that was developed for treating patients with advanced malignancies. Kras^G12D/+^; Trp53^R172H/+^; Pdx-1-Cre (KPC) mice mimic human disease, with similar progression and incidence of metastasis. Treatment of established tumors in KPC mice with INCB057643 increased survival by an average of 55 days, compared to the control group. Moreover, INCB057643 reduced metastatic burden in these mice. KPC mice treated with INCB057643, starting at 4 weeks of age, showed beneficial changes in immune cell populations in the pancreas and liver. Similarly, INCB057643 modified immune cell populations in the pancreas of Kras^G12D/+^; Pdx-1-Cre (KC) mice with pancreatitis, an inflammatory process known to promote pancreatic cancer progression. The data presented here suggest that the bromodomain inhibitor INCB057643 modulates the TME, reducing disease burden in two mouse models of pancreatic cancer. Furthermore, this work suggests that BRD4 may play a role in establishing the TME in the liver, a primary metastatic site for pancreatic cancer.

## 1. Introduction

Pancreatic ductal adenocarcinoma (PDA) is one of the most aggressive cancers, with ~10% survival five years after diagnosis [1]. PDA is expected to become the second leading cause of cancer-related deaths in the United States by 2030, being surpassed only by lung cancer [2]. Significant advances have been made in recent years with Food and Drug Administration (FDA) approval of immunotherapies for solid cancers. However, no significant clinical responses to immunotherapy have been observed in patients with PDA [3], so this disease remains one of the most difficult cancers to treat. Current standard of care chemotherapy for PDA is FOLFIRINOX or the combination of gemcitabine/nab-paclitaxel (Abraxane), which produce only a modest increase in long-term survival [4].

*KRAS* is the most frequently mutated gene in PDA, and *KRAS* mutations are found in 95% of pancreatic cancers [5]. Although genetically engineered mouse (GEM) models have convincingly demonstrated that constitutive activation of *KRAS* alone is sufficient for the initiation and progression of this disease [6,7,8], progression is accelerated when an inflammatory stimulus is added [9]. Other genes known to be altered in PDA include *TP53*, *p16* and *SMAD4*, with rates of modification or suppression between 50–85% [10]. The multiple genes involved in the progression of pancreatic cancer form a complex interactive circuit of pathways that make PDA difficult to treat [11].

Inflammation can promote pancreatic cancer, and pancreatitis, or inflammation of the pancreas, increases the probability of developing pancreatic cancer by 2.7- to 16.5-fold [12,13]. The expression of genes involved in inflammation, cell growth and cancer progression are tightly regulated. This regulation can be implemented by epigenetic modifications, more specifically by chromatin “readers” that detect and “read” specific modifications [14,15,16]. The bromodomain and extra terminal domain (BET) family of protein members read acetylated histones, thus regulating the assembly of chromatin complexes and transcription at specific promoter sites [17]. Epigenetic mechanisms add an additional layer of complexity to the heterogeneity of pancreatic cancer, but the fact that these mechanisms are reversible offers a unique opportunity for therapeutic intervention [18,19,20]. Selective inhibitors of BET proteins have been developed to occupy the binding pockets of these proteins, leading to the release from chromatin and inhibition of downstream signals [21,22,23]. JQ1 was the one of the first BET inhibitors identified [21] and remains the most studied drug in the class. JQ1 has proven anti-inflammatory and anti-cancer activities, even in preclinical models of pancreatic cancer [24,25,26]. Bromodomain inhibitors are a class of drugs that target BET (bromodomain and extra-terminal) proteins, impairing their ability to bind to acetylated lysines and therefore interfering with transcriptional initiation and elongation. BET proteins regulate several genes responsible for cell growth, apoptosis and inflammation. INCB057643 is a new generation, orally bioavailable BET inhibitor that was tested in Phase 1 clinical trials for patients with advanced myelofibrosis [27]. Here we report the effects of INCB057643 in extending survival in a clinically relevant mouse model of pancreatic cancer. Moreover, we show that INCB057643 modulates immune cell populations within the microenvironment of the pancreas.

## 2. Results

### 2.1. INCB057643 Increases Survival in a KPC Murine Model of Pancreatic Cancer

**Figure 1:**
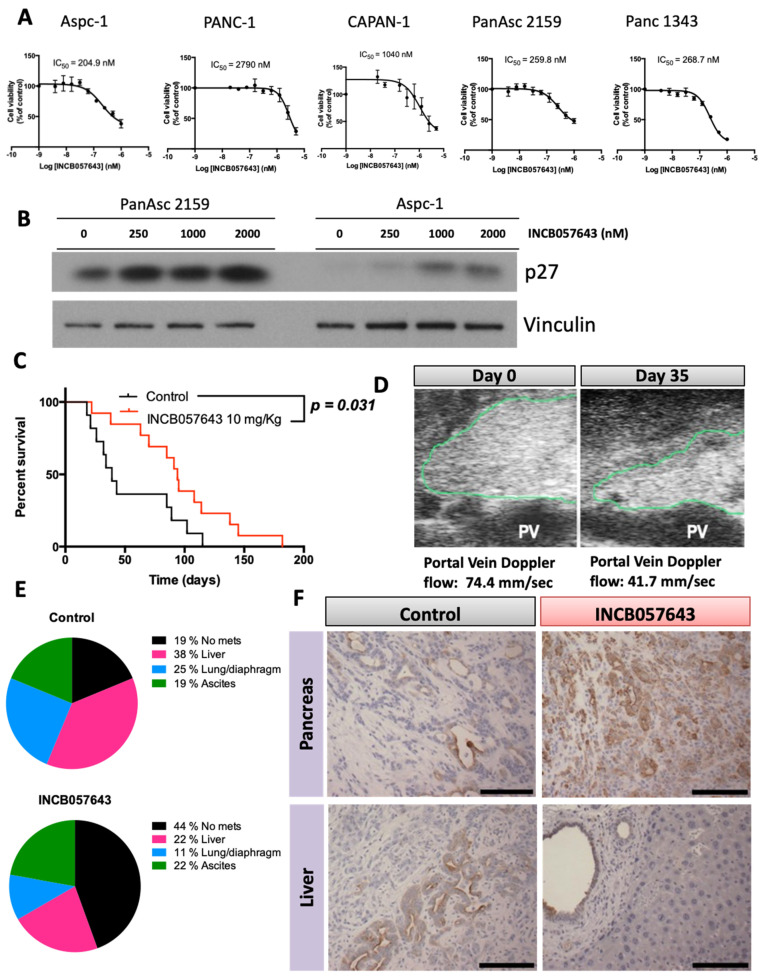
INCB057643 increases survival of KPC mice with pancreas tumors. (**A**) Anti-proliferative effects of INCB057643 in pancreatic cancer cells. IC_50_ values were determined by a MTT assay after 72 h of treatment. The values are the mean ± SE of at least three independent experiments. Pancreatic cancer cell lines: Aspc-1, PANC-1 and CAPAN-1 (human); Panc1342 and PanAsc 2159 (mouse). (**B**) Immunoblots for p27, a regulator of cell cycle. Aspc-1 and PanAsc 2159 were treated with INBC057643 at the indicated concentrations for 24 h. (**C**) Kaplan Meir plot for LSL-Kras^G12D/+^; LSL-Trp53^R172H/+^; Pdx-1-Cre (KPC) mice treated with vehicle (control, *n* = 11) or INCB057643 (*n* = 13). KPC mice, with pancreas tumors detected by palpation and confirmed by ultrasound, were treated with vehicle or INCB065743 (10 mg/kg) by gavage every day for 2 months. Mice that survived longer than 2 months were then gavaged 5 days a week. (**D**) Ultrasound image of the pancreas of a mouse after 35 days of treatment with INCB057643. The green contour indicates the pancreas tumor and PV refers to portal vein; flow rates in the portal vein are listed. (**E**) Metastatic burden observed at the time of necropsy in KPC mice treated with vehicle control or INCB057643 as described above. (**F**) Cytokeratin immunohistochemistry in the pancreas and metastatic livers of control and INCB057643-treated KPC mice. Scale bar 30 µm. Uncropped Western Blot Images are shown in Figures S5 and S6.

BRD4 is widely expressed in all tissues, including the pancreas [28]. Before testing the small molecule inhibitor INCB057643 in our mouse models, we first confirmed that BRD4 is expressed in pancreatic cancer cell lines (Figure S1A,B) and the pancreas of both LSL-Kras^G12D/+^; Pdx-1-Cre (KC) and LSL-Kras^G12D/+^; LSL-Trp53^R172H/+^; Pdx-1-Cre (KPC) mice. The cell lines were derived from pancreatic tumors from KPC mice. Both models express BRD4 in the pancreas, with expression in early lesions (pancreatic intraepithelial neoplasia or PanINs) and visible tumors (Figure S1A). The role of BRD4 in proliferation of cancer cells has been exploited in recent years with several small molecule inhibitors entering clinical trials [27]. INBC057643 was developed as a BRD inhibitor, and it reduced cell proliferation and increased expression of p27, indicative of cell cycle arrest, in several murine and human pancreatic cancer lines (Figure 1A,B), as has been reported for other BRD4 inhibitors [24].

When used to treat established tumors in the KPC mouse model of pancreatic cancer, INCB057643 treatment suppressed tumor growth and reduced the incidence of metastasis (Figure 1C,D). KPC mice develop pancreatic cancer around 22 weeks of age with a high metastatic incidence, similar to the human disease [29]. In this study, KPC mice were monitored by palpation and by ultrasound imaging for the presence of a pancreas tumor. Once a tumor was detected and reached an approximate size of 0.2–0.4 cm, mice were randomized and enrolled on treatment with INCB057643 or vehicle control. Treatment with INCB057643 was done by gavage at 10 mg/kg every day for 60 days. If mice survived longer than 60 days, INCB057643 was administered five days a week until the end of study. INCB057643 treatment prolonged survival, when compared to the control group, from an average of 39 days to 94 days (*p* = 0.031, Figure 1C). Moreover, INCB057643 decreased tumor size when viewed non-invasively using ultrasound, but no change in tumor size at the time of necropsy was observed. Splenic/portal vein blood flow was also lowered, suggesting less deformation of the vessels by the tumor and/or decreased stiffness, a common hallmark of pancreatic cancer (Figure 1D). In pancreatic cancer patients, vascular involvement can determine the feasibility of surgical removal of the tumor [30,31].

INCB057643 also reduced the incidence of metastasis in KPC mice. In control mice only three out of the 11 mice enrolled had no visible metastases at the time of necropsy. In the group treated with INCB057643, eight out of the 13 enrolled mice did not have visible metastases at time of death (Figure 1E, *p* = 0.122). In the control mice with metastases, six had liver metastases, three had ascites and four had lung and/or diaphragm metastases. In mice treated with INCB057643, four had liver metastases, four had ascites and two had lung and/or diaphragm metastases (Figure 1E). Both control and INCB05743 treated mice had metastatic disease in more than one site, six mice in the control group (one mouse with metastatic disease at all sites, three with liver metastases and ascites, and two with liver and lung metastases) and four in the INCB057643 group (one mouse with metastasis in all sites, one mouse with lung metastases and ascites, and two mice with liver metastases and ascites).

Tumors in vehicle treated mice showed a less differentiated architecture, with a higher percentage of stromal cells and fewer tumor cells (cytokeratin 19 positive cells). In contrast, tumors of mice treated with INCB057643 were more differentiated with less stromal deposition (Figure 1F). Cytokeratin 19 staining of the liver in mice that had visible metastatic disease at the time of necropsy showed that mice treated with INCB057643 had lesions with more defined architecture and did not invade into the surrounding tissue (some PanINs are still visible) when compared with vehicle treated mice (disorganized structure and invasion into surrounding tissue).

### 2.2. The Bromodomain Inhibitor INCB057643 Changes Immune Populations in the Pancreas and Liver

Immunomodulatory functions of BRD4 have been described for some cancers [28,32,33,34] and BRD4 inhibitors epigenetically regulate selected immune cells, such as CD8 T cells [32]. In order to understand the immune regulation with INCB057463, KPC mice were treated (10 mg/kg) from weeks 4–20 for 5 days/week, with a two day/week holiday. At the 20 week end-point, pancreas, liver and spleen were collected and analyzed by flow cytometry. No changes in the weight of pancreas, liver or spleen were observed (Figure S2).

**Figure 2:**
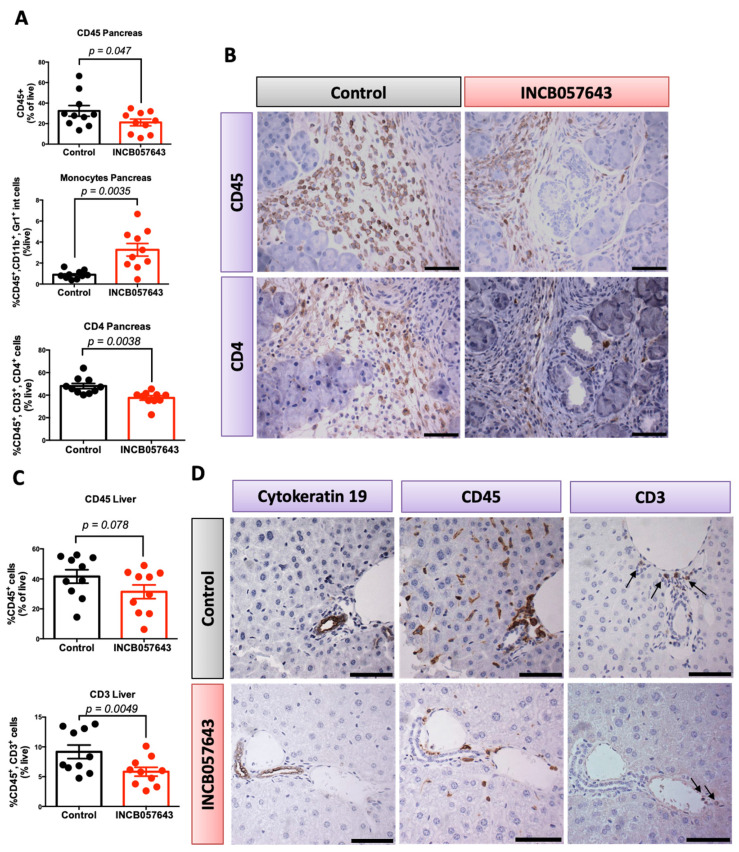
INCB057643 changes the inflammatory microenvironment in the pancreas and liver of KPC mice. KPC mice were treated with INCB057643 5 times a week for 16 weeks, starting at 4 weeks of age. Blood, pancreas, liver, lung and spleen were collected. *n* = 10 mice/group. CD45^+^ immune cells, CD45^+^, CD11b^+^, Gr1^+^low cells (monocytes), CD45^+^, CD3^+^ (CD3 T cells), and CD45^+^, CD3^+^, CD4^+^ (CD4 T cells) were analyzed by flow cytometry in the pancreas (**A**) and liver (**C**). Flow cytometry results were confirmed by immunohistochemistry in both pancreas (**B**) and liver (**D**). Arrows on panel (**D**) indicate positive CD3 T cells. Scale bar 30 µm.

Mice treated with INCB057643 had a significantly lower infiltration of total immune cells (CD45 positive) and CD4 T cells (CD45^+^, CD3^+^, CD4^+^) in the pancreas (*p* = 0.047 and *p* = 0.0038, respectively, Figure 2A). Immune cell infiltration into the pancreas is an established hallmark of pancreatic cancer and promotes tumor growth [35]. Moreover, a significant increase in monocytes (CD45^+^, CD11b^+^, Gr1^low^; Figure 2A) was observed. The role of these monocytes in pancreas cancer is not well established, with some contradicting reports on the impact on overall survival when the lymphocyte to monocyte ratio in the blood of pancreatic cancer patients was evaluated [36,37,38]. No changes in macrophages (CD45^+^, CD11b^+^, Gr1^−^) or total T cells (CD45^+^, CD3^+^) infiltrating into the pancreas were observed (Figure S2A). The changes in CD45 and CD4 were confirmed by immunohistochemistry in pancreas sections (Figure 2B).

In agreement with the changes in the immune cell populations, protein extracts of pancreas showed a trend toward decreased expression of COX-2 (Figure S2D), although not statistically significant, possibly due to the variation between animals. COX-2 is a main regulator of inflammation, which contributes to disease progression and skewing of immune cells toward a tumor-promoting phenotype [39]. A trend toward increased E-cadherin in mice treated with INCB057643 was also observed (Figure S2D), consistent with the more differentiated phenotype observed in the survival study. E-Cadherin is a junction protein, required to maintain an epithelial phenotype [40], and increases in this marker, although not statistically significant, suggest that INCB057643 can induce cell differentiation.

Because of the decrease in metastasis observed in the survival study with INCB057643, livers were also analyzed by flow cytometry. The liver is the most common organ colonized by pancreatic cancer cells and immune cells are known to contribute to this process [41,42]. Mice treated with INCB057643 had a significant decrease in the number of T cells (CD45^+^, CD3^+^) infiltrating into the liver (*p* = 0.0049), with a marked reduction in total immune cells (*p* = 0.078) (Figure 2C). No changes were observed in the number of CD4 and CD8 T cells infiltrating into the liver of treated mice when compared with the controls (Figure S2C). The changes observed by flow cytometry were confirmed by immunohistochemistry; CD45 and CD3 cells accumulate in areas where PanINs are present (Cytokeratin 19 positive, Figure 2D).

Liver protein extracts showed a trend toward a decrease in the expression of COX-2 (*p* = 0.110), a regulator of inflammation, and a decrease in CD206 (*p* = 0.245). Macrophages are abundant in the liver, and CD206 is a marker of tumor-promoting macrophages [41]. The percentage of macrophages infiltrating into the liver at 20 weeks did not change in mice treated with INCB057643 (Figure S2C,E). These data suggest that INCB057643 can epigenetically change macrophages in the liver and reduce their ability to support tumor growth.

### 2.3. INBC057643 Modulates Immunosuppressive Pathways in CD4 T Cells and Macrophages

Recently, immunotherapy has emerged to play a central role in treatment of several solid cancers. However, pancreatic cancer patients have not benefited from these efforts. Pancreatic cancer has a dense immunosuppressive tumor microenvironment where regulatory T cells and macrophages abound [43]. Epigenetic regulation of T cells and macrophages is an important next generation therapeutic target that could enhance responses to immunotherapy [44]. To understand the effects of INCB057643 on Tregs and macrophages, levels of FOXP3 and PD-L1 were analyzed in 20-week old KPC mice.

**Figure 3:**
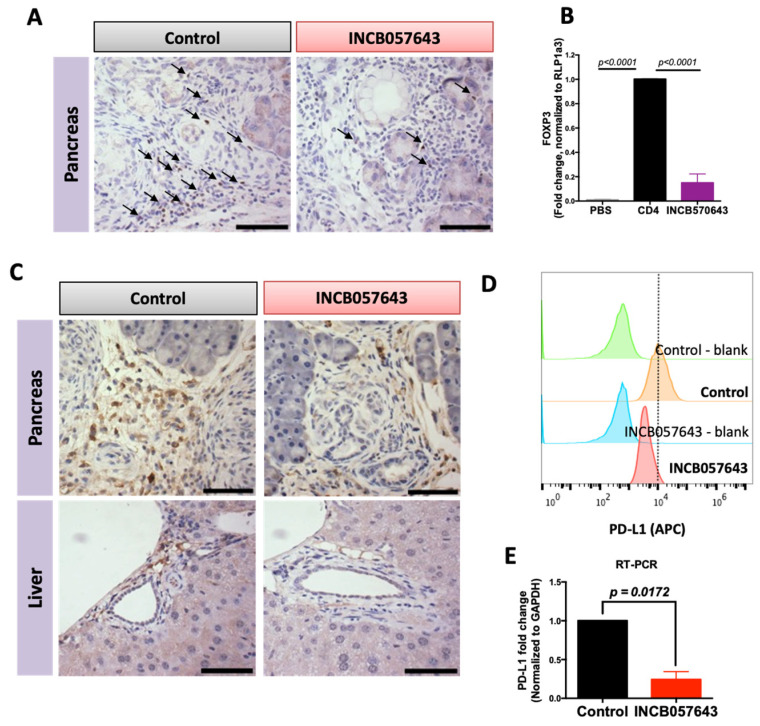
INCB057643 reduces the expression of FOXP3 in CD4 T cells and PD-L1 expression in macrophages. Immunohistochemistry for FOXP3 (**A**) or PD-L1 (**C**) in pancreas and liver of KPC mice treated with vehicle control or INCB057643 for 16 weeks. Scale bar = 30 µm. (**B**) CD4 T cells were isolated from a spleen of a wild type mouse using negative magnetic bead selection. CD4 T cells were plated with anti-CD3 (or PBS, a control for non-stimulated T cells), anti-CD28, IL-2 and TGF-β for 24 h prior to adding INCB057643 (100 nM) for 4 days. CD4 T cells were collected and levels of FOXP3 were determined by PCR. *p* < 0.001 vs. control CD4 cells in 2 independent experiments. (**D**) RAW 264.7 macrophage-like cells were treated with conditioned media from PanAsc 2159 cells; representative histograms for levels of PD-L1 analyzed by flow cytometry after treatment with 0.5 μM INCB057643 for 24 h are shown (*n* = 2) and confirmed by RT-PCR (*n* = 3) (**E**).

FOXP3, a nuclear protein, is the main regulator of T regulatory immunosuppressive function [45]. In cancer, increased numbers of Treg cells, and therefore high FOXP3 expression, is associated with poor prognosis [43]. Treatment with INCB057643 for 16 weeks decreased the total number of CD4 T cells in the pancreas of KPC mice and the total number of CD3 T cells in the liver (Figure 2A,C). These decreases were accompanied by a decrease in the expression of FOXP3 in the pancreas, as observed by immunohistochemistry (Figure 3A). To confirm that the decrease in FOXP3 expression is not simply a consequence of the reduced number of T cells, an in vitro assay was established to determine the effects of INCB057643 on the expression of FOXP3 in T cells. CD4 T cells isolated from the spleen of a wild type C57BL/6 mouse and stimulated with anti-CD3, CD28, IL-2 and TGF-β to induce the expression of FOXP3 were treated with INCB057643 (100 nM) for four days. INCB057643 reduced the expression of FOXP3 by 85% (*p* < 0.0001) when compared to control CD4 T cells (Figure 3B). These results suggest that INCB057643 reduces the immunosuppressive phenotype in CD4 T cells.

BRD4 regulates PD-L1 transcription in ovarian cancer cells, leading to a decrease in the expression of PD-L1 [46]. The PD-L1/PD-1 pathway is a T cell inhibitory pathway that can be hijacked by cancer cells to promote an immunosuppressive microenvironment. Both PD-L1 and PD-1 can be expressed by antigen presenting cells, such as macrophages, thereby reducing their ability to activate T cells. Because other bromodomain inhibitors, such as JQ1 and I-BET, modulate inflammatory properties of macrophages [47], we hypothesized that INCB057643 can regulate the expression of PD-L1 in macrophages. In KPC mice treated with INCB057643 for 16 weeks, PD-L1 expression was reduced in the pancreas and liver, when compared with vehicle treated mice (Figure 3C). In order to elucidate if the reduced expression in PD-L1 was regulated in macrophages, RAW 264.7 cells were stimulated with conditioned media from murine PanAsc 2159 pancreatic cancer cells and treated with INCB057643 at 500 nM for 24 h. Levels of PD-L1 were analyzed by flow cytometry and RT-PCR. INCB057643 reduced the expression of PD-L1 in RAW 264.7 cells (Figure 3D,E and Figure S3A). In both human (Aspc-1) and murine (PanAsc 2159) pancreatic cancer cells, INCB057643 reduced the expression of PD-L1, but the reduction in MFI was smaller in the cancer cells than in the RAW264.7 cells (Figure S3B). A significant reduction in the levels of PD-L1 mRNA expression in PanAsc2159 cells treated with INCB057643 was confirmed by RT-PCR (Figure S3C). Thus, INCB057643 regulates the immunosuppressive properties of the tumor microenvironment in pancreatic cancer. INCB057643 modulates both macrophages and CD4 T cells, by reducing expression of PD-L1 and FOXP3, respectively.

### 2.4. Bromodomain Inhibitor INCB057643 Attenuates Pancreatitis and Immune Infiltration in KC Mice

Pancreatitis, or inflammation of the pancreas, is a painful, often recurrent, and sometimes lethal medical disorder that has increased in incidence in the past decade [12]. Pancreatitis increases the risk for pancreatic cancer by 2.7- to 16.5-fold, and patients with hereditary pancreatitis have a 40–55% lifetime risk of developing pancreatic cancer [12,13]. In the commonly used LSL-Kras^G12D/+^; Pdx-1-Cre (KC) mouse model [48] with an activating *Kras* mutation targeted to the pancreas, pancreatitis accelerates the development of pancreatic cancer [49,50]. Increased infiltration of immune cells is found in both human and murine pancreatitis [51].

**Figure 4:**
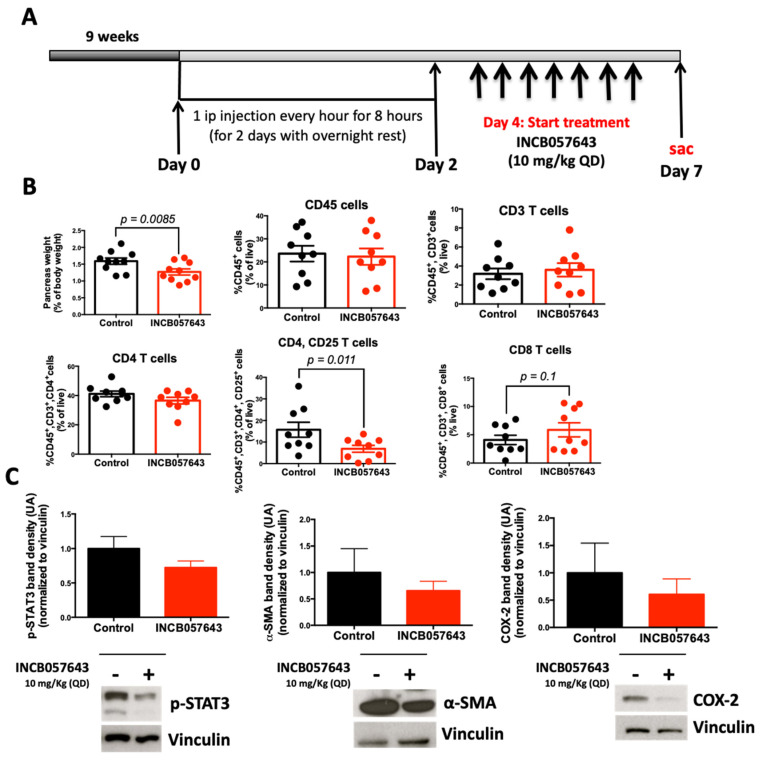
INCB057643 changes the inflammatory microenvironment in the pancreas of KC mice. LSL-Kras^G12D/+^; Pdx-1-Cre (KC) mice were treated with INCB057643 7 times a week, for 1 week, starting at 9 weeks of age. Blood, pancreas, and spleen were collected. (**A**) Experimental design, *n* = 9-10 mice/cohort. (**B**) CD45^+^ immune cells, CD45^+^, CD3^+^ (CD3 T cells), CD45^+^, CD3^+^, CD4^+^ (CD4 T cells), CD45^+^, CD3^+^, CD4^+^, CD25^+^ (activated CD4 T cells), and CD45^+^, CD3^+^, CD8^+^ (CD8 T cells) were analyzed by flow cytometry in the pancreas. (**C**) Levels of COX2, p-STAT3 and α-SMA in pancreas extracts were determined by western blot. For quantitation of the blots, INCB057643-treated samples were normalized to controls. Graph bar represents 6–10 mice/group. Uncropped Western Blot Images are shown in Figure S9.

To test if INCB057643 can be used to mitigate pancreatitis, KC mice were stimulated with cearulein to induce acute pancreatitis (Figure 4A). INCB057643 was administered by oral gavage (10 mg/kg), starting the day after cearulein stimulation, for 7 days. Flow cytometry was used to analyze immune cell populations in the pancreas and spleen. INCB057643 treatment significantly reduced the weight of the pancreas (*p* = 0.0085), this reduction was accompanied by a reduction in activated CD4 T cells (CD45, CD3, CD4, CD25, *p* = 0.011; Figure 4B). Pancreas extracts showed a 30% and 40% reduction in the expression of p-STAT3 and COX2 proteins respectively (Figure 4C). Additionally, INCB057643 reduced by 35% the expression of α-SMA, a marker of activated stellate cells responsible for extra-cellular deposition of matrix and the desmoplastic reaction in pancreatic cancer. The changes in histology and α-SMA expression were confirmed by histology and IHC (Figure S4).

## 3. Discussion

Bromodomain inhibitors have shown promise in multiple preclinical mouse models of cancer, including pancreatic cancer [24,26,52]. However, clinical trials have not reproduced these exciting results, mostly due to pharmacokinetic/pharmacodynamic challenges with these inhibitors [21]. In this study, a new bromodomain inhibitor, INCB057643, has growth inhibitory and immunomodulatory properties in pancreatic cancer cells and immune cells, respectively. These important properties of INCB057643 are reflected in the changes in immune cells infiltrating into the pancreas and the liver and in the alterations in FOXP3 and PD-L1 expression. Most importantly, INCB057643 extends the survival of KPC mice bearing pancreatic tumors and reduces the incidence of metastatic disease.

Immunomodulation by INCB057643 is consistent with similar findings for other bromodomain inhibitors [28]. In pancreatic cancer the functions of the stroma in promoting carcinogenesis are well established and are considered a focal point for therapeutic targeting [53,54]. Here we report the effects of INCB057643 in Tregs and macrophages, two important populations for promoting and maintaining an immunosuppressive microenvironment. Tregs accumulate in mouse and human PanINs and PDAC; Treg frequency correlates with tumor metastasis and poor prognosis in patients with PDAC [55]. Treg depletion in a model of implanted PDAC led to a CD8-mediated anti-tumor immune response [43,55]. INCB057643 reduced the expression of FOXP3 in the pancreas of KPC mice and in an in vitro experiment. FOXP3 is a transcription factor that regulates the function of CD4 Treg cells. The molecular mechanisms regulating FOXP3 are complex and consist of finely tuned transcriptional and epigenetic events [56]. Although the role of BRD4 and inhibition of FOXP3 were previously unknown, our data suggest that BRDs can regulate the expression of FOXP3.

The PD-1/PD-L1 pathway has been heavily targeted as a treatment for cancer, with several inhibitors approved by the FDA for the treatment of KRAS-driven cancers, including colon and lung cancers [57]. Pancreatic cancer, however, has not benefited from PD-1/PD-L1 blockade, mostly due to the presence of a dense stromal reaction that prevents delivery of these molecules to the tumor [53]. Epigenetic modulation has been considered a promising strategy to increase responsiveness or bypass resistance to immunotherapy [44,58]. We show that INCB057643 can reduce the expression of PD-L1 in macrophages (Figure 3), a predominant immunosuppressive population in pancreatic cancer [43]. Although no changes in the percentage of CD8 T cells were observed by flow cytometry (data not shown), future studies will investigate if the decrease in PD-L1 is accompanied by enhanced activity of CD8, leading to the increase in survival and reduction in metastatic disease observed with INCB05743 treatment (Figure 1). Reports that unique neoantigens in pancreatic cancer patients correlate with increased survival [59] might point to a unique opportunity to modulate antigen presentation through epigenetic regulation [44,58] with BRD inhibitors.

## 4. Materials and Methods

### 4.1. Drugs

INCB057643 (>98% purity) was provided by Incyte Corporation (Wilmington, DE, USA).

### 4.2. Cell Culture and Reagents

Aspc-1, PANC-1 and CAPAN-1 cells purchased from ATCC (Manassas, VA, USA) were grown in RPMI with 10% fetal bovine serum (FBS), DMEM with 10% FBS, and Iscove’s Modified Dulbecco’s with 20% FBS, respectively. The mouse pancreatic cancer cell lines PanAsc 2159 and Panc 1343 were generated from KPC mice as previously described [60] and grown in DMEM with 10% FBS. RAW 264.7 mouse macrophage-like cells (from ATCC) were cultured in DMEM supplemented with 10% FBS. All cell lines were supplemented with 100 units/mL penicillin/streptomycin. Cells were cultured at 37 °C in a humidified incubator with 5% CO_2_.

### 4.3. Cell Viability

Cells were seeded in 96-well plates at the following optimized densities: PanAsc 2159 (2500 cells/well), Panc 1343 (1500 cells/well), Aspc-1 (2000 cells/well), PANC-1 (7500 cells/well) and CAPAN-1 (12,000 cells/well). Cells were allowed to grow for 12 h before compounds were added in a series of dilutions. After 72 h, cells were incubated with MTT (3-[4,5-dimethylthiazol-2-yl]-2,5-diphenyltetrazolium bromide; thiazolyl blue; Sigma-Aldrich (St. Louis, MO, USA) for 4 h before the supernatant was removed and developing solution (0.04 N HCl in isopropanol) was added. Plates were read at 630–570 nm.

### 4.4. Western Blotting

Treated cells were lysed in RIPA buffer (1 M Tris-Cl, pH 7.4, 0.5 M EDTA, 5 M NaCl, 1% triton-X, 25 mM deoxycholic acid, 0.1% SDS) with protease inhibitors (PMSF, aprotinin and leupeptin). Pancreata were homogenized in EBC buffer (1M Tris pH 8, 5 M NaCl) with the same protease inhibitors and 10% NP-40 and then incubated on ice for 30 min. Protein concentrations were determined by the BCA assay (St. Louis, MO, USA). Proteins were resolved by SDS-PAGE, transferred to a nitrocellulose membrane and analyzed with the following antibodies: p27^KIP1^, COX-2, p-STAT3 and vinculin (all from Cell Signaling, Danvers, MA, USA), BRD4 (Abcam, Cambridge, MA, USA) and E-Cadherin (Santa Cruz, Dallas, TX, USA); secondary antibodies were purchased from Santa Cruz and Cell Signaling. ImageJ (NIH, Bethesda, MD, USA) was used for the quantification of the immunoblots, and results were plotted and statistically analyzed using Prism 6. All images shown are representative of 2–3 independent experiments.

### 4.5. In Vivo Experiments

KC mice (LSL-Kras^G12D/+^; Pdx-1-Cre) on a C57BL/6 background were obtained by interbreeding male LSL-Kras^G12D/+^; Pdx-1-Cre and female Pdx-1-Cre mice. Genomic DNA was extracted from tail snips using the Extract-N-Amp tissue PCR kit (Sigma) and genotyped [29,48]. Four week-old KC mice were randomized and fed powered 5002 rodent chow. Nine week-old KC mice were treated with INCB057643 (10 mg/kg). INCB057643 was dissolved in 5% (*v*/*v*) DMSO and then added to a solution of 10% tween in saline and administered by gavage. Cearulein were administered by intraperitoneal injection, and the pancreas, spleen and blood were collected 24 h after the last INCB057643 administration. Cearulein (Sigma) was administered at 75 μg/kg every hour for 8 h for 2 consecutive days, with an overnight rest.

KPC mice (LSL-Kras^G12D/+^; LSL-Trp53^R172H/+^; Pdx-1-Cre) on a C57BL/6 background were obtained by interbreeding male LSL-Kras^G12D/+^; Pdx-1-Cre and female Trp53^R172H/+^; Pdx-1-Cre mice. Genomic DNA was extracted from tail snips using the Extract-N-Amp tissue PCR kit (Sigma) and genotyped. Four week-old KPC mice were randomized and fed powered 5002 rodent chow and monitored for disease. Monitoring was done by palpating the abdominal cavity for abnormal growths twice a week after 16 weeks of age; the presence of tumors was confirmed by ultrasound.

### 4.6. Flow Cytometry

One third of the pancreas and spleen removed from KC or KPC mice was minced and incubated separately in digestion media consisting of collagenase (300 U/mL, Sigma), dispase (1 U/mL, Worthington, Lakewood, NJ, USA), and DNAse (2 U/mL, Calbiochem, Burlington, MA, USA) for 30 min at 37 °C with stirring. Cells were then passed through a 40 μm cell strainer (BD Falcon, Glendale, AZ, USA), and RBC eliminated with lysing solution (eBioscience, San Diego, CA, USA). Single cells were resuspended in a solution of PBS/0.5% BSA/0.1% azide and stained 30 min at 4 °C with the following antibodies: CD45-VioGreen (30F11, Miltenyi, Bergisch Gladbach, Germany, 10:100), Gr-1-PE (RB6-8C5, Miltenyi, 10:100), CD11b-FITC (M1/70.15.11.5, Miltenyi, 10:100), CD19-APC (1D3/CD19, Biolegend, San Diego, CA, USA, 1:100), B220-PerCP-Cy5.5 (RA3-6B2, Biolegend, 1:100), CD3-PE (145-2C11, Biolegend, 1:100), CD4-FITC (Gk1.5, Miltenyi, 10:100), CD8-APC (53-6.7, Biolegend, 1:100), CD25-PE.Cy7 (EBioscience, 1:100) and 5 μg/mL anti-mouse CD16/CD32 antibody (Biolegend) to reduce antibody binding to Fc receptors. Propidium iodide staining was used to exclude dead cells. Cells were analyzed using an LSR II-DIVA 6,2 software (BD) with three laser sources (488 nm, 633 nm, 407 nm) and FlowJo x.10.0.7r2 software (Tree Star, Ashland, OR, USA).

For PD-L1 staining, RAW 264.7 cells were plated at 50,000 cells/mL in 6 well plates for 24 h before adding conditioned media from PanAsc 2159 cells. RAW 264.7 cells were cultured for an additional 24 h with PanAsc 2159 conditioned media and INCB057643. PanAsc 2159 or Aspc-1 cells were seeded at 2000 cell/mL in 6 well plates for 24 h before adding INCB057643 for 72 additional hours. RAW 264.7, PanAsc 2159 or Aspc-1 cells were collected and resuspended in a solution of PBS/0.5% BSA/0.1% azide and stained for 30 min at 4 °C with anti-PD-L1-APC (10F.9G2, Biolegend, 1:100) and 5 μg/mL anti-mouse CD16/CD32 antibody (Biolegend) to reduce antibody binding to Fc receptors. Cells were analyzed using a BD Accuri C6 (BD) with two laser sources (488 nm and 640 nm) and FlowJo x.10.0.7r2 software (Tree Star).

### 4.7. Immunohistochemistry

One third of the pancreas and spleen removed from KC or KPC mice was fixed in 10% phosphate-buffered formalin for at least 48 h, embedded in paraffin blocks and sectioned (5–6 µm). Hydrogen peroxide was used to quench endogenous peroxidase activity. Sections were immunostained with antibodies raised against Cytokeratin 19 (1:400, Abcam), FOXP3 (1:50, FJK-16s, EBioscience), PD-L1 (1:50, MIH6, Abcam), CD4 (1:40, Gk1.5, Biolegend), or CD45 (1:100, 30-F11, BD Pharmingen, San Diego, CA, USA) and visualized with biotinylated anti-rabbit or anti-rat secondary antibodies (Cell Signaling or Vector Labs, Burlingame, CA, USA). Signal was detected using a DAB substrate (Cell Signaling) following the manufacturer’s recommendations. Sections were counterstained with hematoxylin (Vector Labs). For RT-PCR cells were collected and RNA was extracted using TRIzol (Thermo Fisher, Grand Island, NY, USA) following the manufacturer’s instructions. Two micrograms of RNA were reverse transcribed, and 1 μL of complementary DNA from this reaction was added to 12.5 μL of iQ SYBRGREEN Supermix (Bio-Rad, Hercules, CA, USA), 1 μL of validated RT2 quantitative PCR (qPCR) PD-L1 (F: 5′-TGC GGA CTA CAA GCG AAT CAC G-3′; R: 5′-CTC AGC TTC TGG ATA ACC CTC G-3′), GAPDH (F: 5′-GGAGCGAGATCCCTCCAAAAT-3′; R: 5′-GGCTGTTGTCATACTTCTCATGG-3′).

### 4.8. CD4-FOXP3 T Cell Treatment

From a single cell suspension of splenocytes from a wild type mouse, 10^7^ cells were incubated in 100 μL of Mojo Buffer (Biolegend) with 10 μL of pre-diluted Biotin-antibody cocktail mix for 15 min, followed by 10 μL of pre-diluted streptavidin nanobeads for an additional 15 min on ice (Biolegend MojoSort^TM^ CD4 T cell isolation kit). The mixture was then placed on the column in the magnetic separator and washed with 3 mL of Mojo Buffer. Isolated CD4 T cells were plated in a 24 well plate at 10^6^ mL/well in RPMI media supplemented with 10% FBS, anti-mouse CD28 (clone 37.51, 3 μg/mL, Biolegend), recombinant IL-2 (5 ng/mL, Biolegend), and recombinant human TGFβ (5 ng/mL, R&D). Plates were coated with anti-mouse CD3ε (clone 145-2C11, 3 μg/mL, Biolegend) over night at 4 °C. After 24 h, INCB057643 was added at 100 nM and CD4 T cells were cultured for an additional 4 days. On day 5 cells were collected and RNA was extracted using TRIzol (Thermo Fisher, Grand Island, NY, USA) following the manufacturer’s instructions. Two micrograms of RNA were reverse transcribed, and 1 μL of complementary DNA from this reaction was added to 12.5 μL of Bio-Rad iQ SYBRGREEN Supermix, 1 μL of validated RT2 quantitative PCR (qPCR) FOXP3 (F: 5′-CTCGTCTGAAGGCAGAGTCA-3′; R: 5′-TGGCAGAGAGGTATTGAGGG-3′) or RLP13A (F: 5′-GTTGCCTTCACA- GCGTA-3′; R: 5′-AGATGGCGGAGGTGCAG-3) primers and DNAse-free water for real-time qPCR. All expression data were normalized using RLP13A as the housekeeping control.

### 4.9. Ultrasound

Anesthesia was induced using 2–4% isofluorane in oxygen at 0.8–2 L/min in a chamber and then maintained using 1.5–2% isofluorane in oxygen at 0.8 L/min using a nosecone. Animals were kept on a heating pad or a heated platform while under anesthesia. Fur was removed from the ventral area using a chemical depilitory (Nair, Church & Dwight Co., Inc., Ewing, NJ, USA) immediately prior to imaging. A Vevo 2100 (Fujifilm VisualSonics, Toronto, ON, Canada) high-frequency ultrasound with a MS550D 40MHz transducer was used to image pancreas tumors qualitatively in B-mode and portal vein/splenic vein flow using pulsed-wave Doppler.

### 4.10. Statistical Analyses

Results are described as mean ± standard error. Data were analyzed by one-way analysis of variance (ANOVA) followed by a Dunnett test (Prism 6) when more than two sets of data were compared. Paired *t* tests were used when two data sets were compared. All *p* values are two-sided; *p* < 0.05 was considered significant.

## 5. Conclusions

INCB057643 displays a multifaceted mode of action, interfering with multiple signaling and transcriptional nodes in both cancer cells and the tumor microenvironment. Drugs that target multiple cell populations are usually not considered beneficial, for the fear of off-target effects [61]. However, in tissues with a complex environment, such as pancreatic cancer with multiple interactions between cells and matrix, this multifunctionality provides important benefits [61]. These experimental results show the advantage of using a multi-targeting/modulating drug, such as INCB057643, in pancreatic cancer, a disease often resistant to standard treatments.

## Data Availability

The data presented in this study and not included within the Supplementary Materials are available on request from the corresponding author. The data are not publicly available due agreements with Incyte regarding the use of INCB057643.

## Supplementary Materials

The following are available online at https://www.mdpi.com/2072-6694/13/1/96/s1, Figure S1. BRD4 expression in pancreas and pancreatic cancer cell lines, Figure S2. INCB057643 changes the inflammatory microenvironment in the pancreas and liver of KPC mice, Figure S3. INCB057643 changes expression of PD-L1, Figure S4. INCB057643 changes the expression of α-SMA in pancreas of KC mice, Figure S5. Uncropped Western Blots for PanAsc 2159 and Aspc-1 experiments, Figure 1 and Figure S1, Figure S6. Uncropped Western Blots for PANC-1 and Panc 1343 experiments, Figure 1 and Figure S1, Figure S7. Uncropped Western Blots for pancreas extracts for Figure S2, Figure S8. Uncropped Western Blots for Liver for Figure S2, Figure S9. Uncropped Western Blots for Figure 4 .
