# Supplementary material for: The Bromodomain Inhibitor, INCB057643, Targets Both Cancer Cells and the Tumor Microenvironment in Two Preclinical Models of Pancreatic Cancer
Source: Cancers (Basel). 2020 Dec 30;13(1):96. doi: 10.3390/cancers13010096 (PMC7794921; doi:10.3390/cancers13010096)
Supplement: Supplementary file 1 [file cancers-13-00096-s001.pdf]

Supplementary Materials: The Bromodomain Inhibitor, INCB057643, Targets both Cancer Cells and the Tumor Micro-environment in two Preclinical Models of Pancreatic Cancer

Ana S. Leal, Phillip Liu, Teresa Krieger-Burke, Bruce Ruggeri and Karen T. Liby

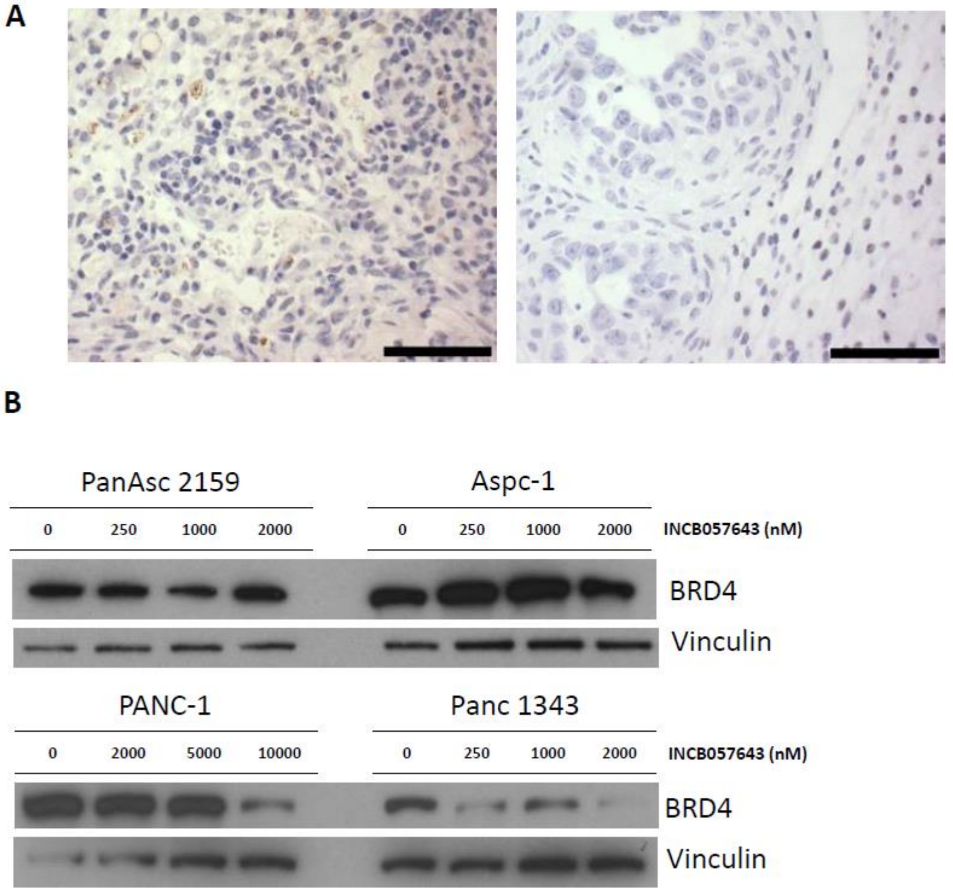

Figure S1. BRD4 expression in pancreas and pancreatic cancer cell lines. (**A**) BRD4 expression in the pancreas of KPC mice with pancreatic tumors. (**B**) BRD4 is expressed in both human (Aspc-1 and PANC-1) and murine (PanAsc 2159 and Panc 1343) pancreatic cancer cell lines. Uncropped Western Blot Images in Figure S5 and Figure S6.

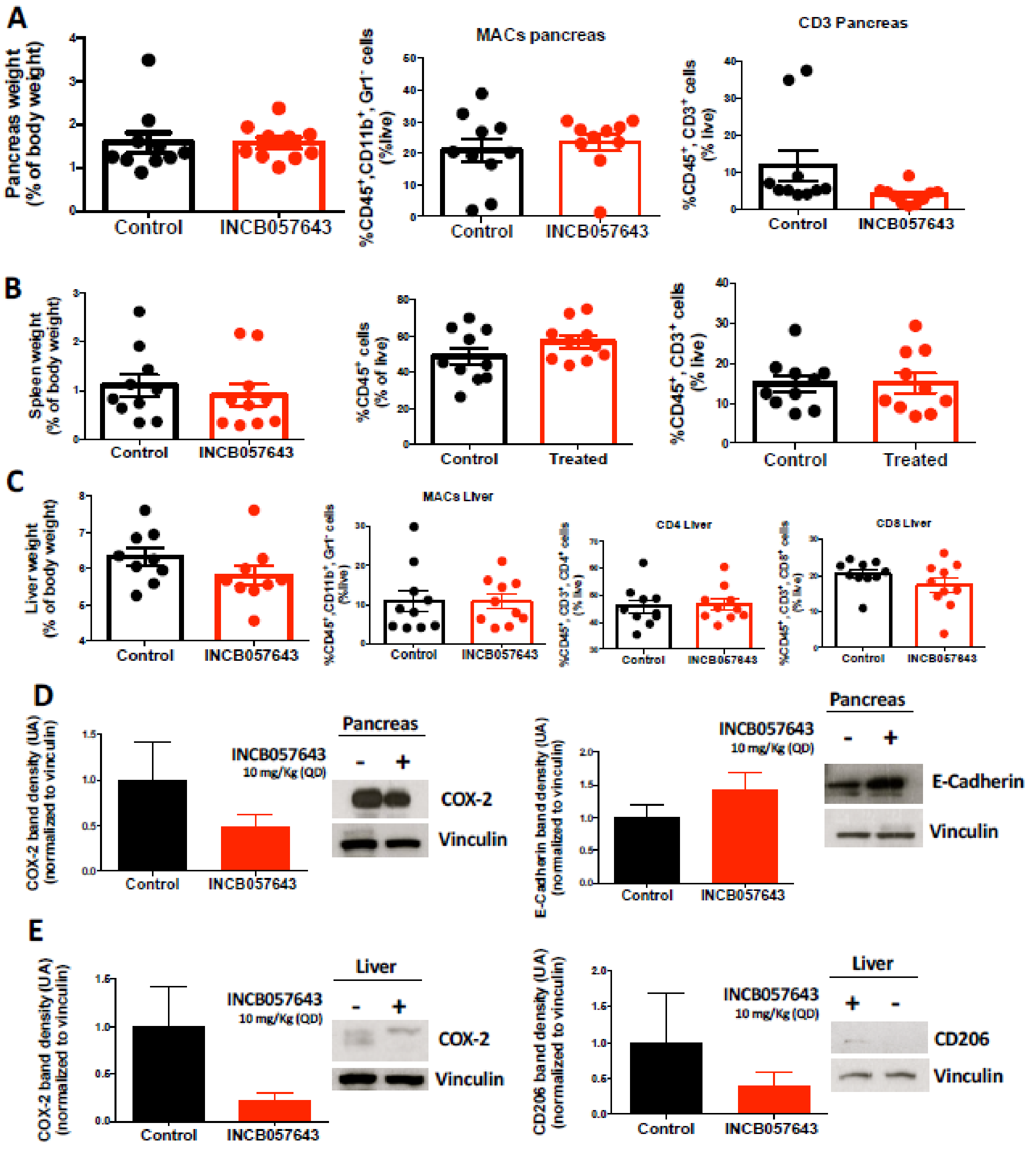

Figure S2. INCB057643 changes the inflammatory microenvironment in the pancreas and liver of KPC mice. LSL-Kras$^{G12D/+}$; LSL-Trp53$^{R172H/+}$; Pdx-1-Cre (KPC) mice were treated with INCB057643 5 times a week, for 16 weeks, starting at 4 weeks of age. Blood, pancreas, liver, lung and spleen were collected. *n* = 10 mice/cohort. (**A**) Levels of CD45$^+$ immune cells, CD45$^+$, CD11b$^+$, Gr1$^-$ cells (MACs = macrophages), CD45$^+$, CD3$^+$ (CD3 T cells), CD45$^+$, CD3$^+$, CD4$^+$ (CD4 T cells) and CD45$^+$, CD3$^+$, CD8$^+$ (CD8 T cells), levels were analyzed by flow cytometry in the pancreas (**A**), spleen (**B**) and liver (**C**). Levels of COX-2, CD206 and E-Cadherin in pancreata (**D**) and liver (**E**) extracts were determined by western blot. For quantitation of the blots, INCB057643-treated samples were normalized to controls. Graph represents 10 mice/group. Uncropped Western Blot Images in Figure S7 and Figure S8.

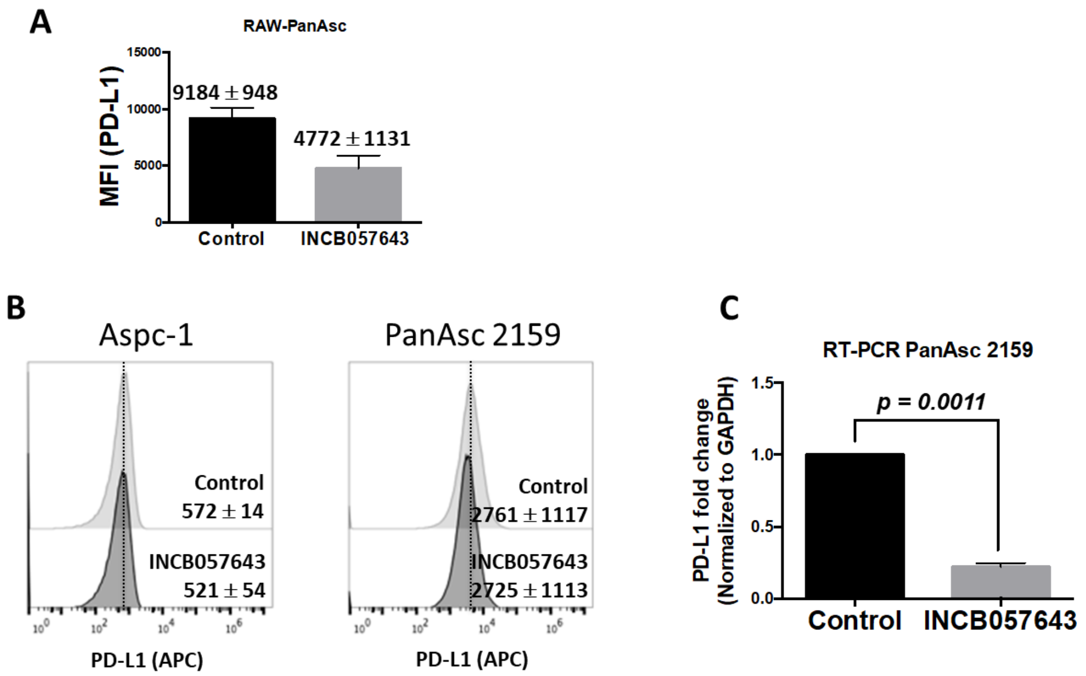

Figure S3. INCB057643 changes expression of PD-L1. (**A**) Mean fluorescence intensity (MFI) of RAW 264.7 macrophage-like cells treated with conditioned media from PanAsc 2159 and INCB057643 (500 nM) for 24 hours (*n* = 2). (**B**) Levels of PD-L1 in Aspc-1 and PanAsc 2159 pancreatic cancer cells treated for 72 hours with INCB057643 (1000 nM) for 72 hours. Representative histograms for 3 independent experiments. Values shown are the mean ±SE of MFI. (**C**) Flow cytometry results were confirmed by RT-PCR (*n* = 3).

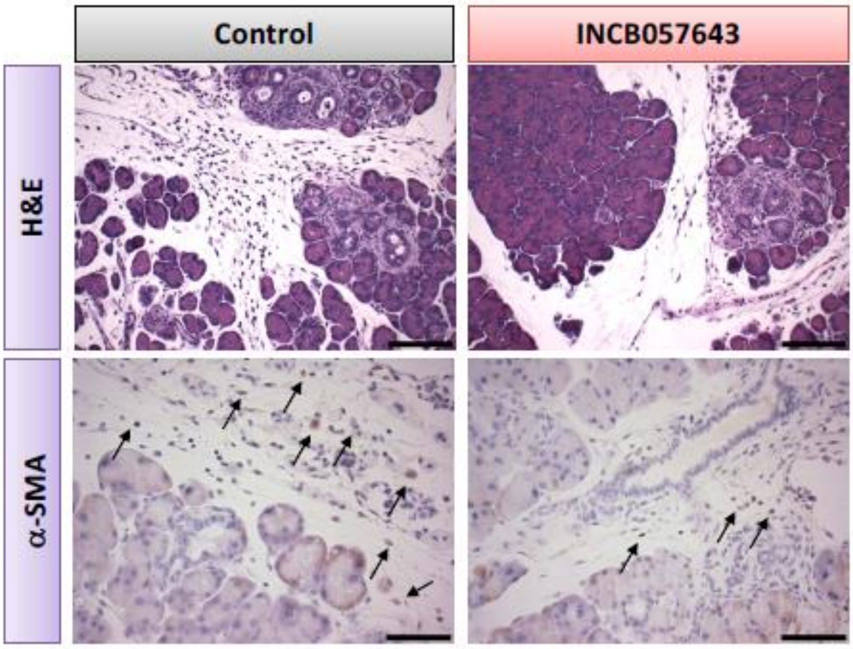

Figure S4. INCB057643 changes the expression of α-SMA in pancreas of KC mice. LSL-Kras$^{G12D/+}$; Pdx-1-Cre (KC) mice were treated with INCB057643 7 times a week, for 1 week, starting at 9 weeks of age. Representative images for H&E and a-SMA. Scale bar for H&E 60 µm and 30 µm for α-SMA.

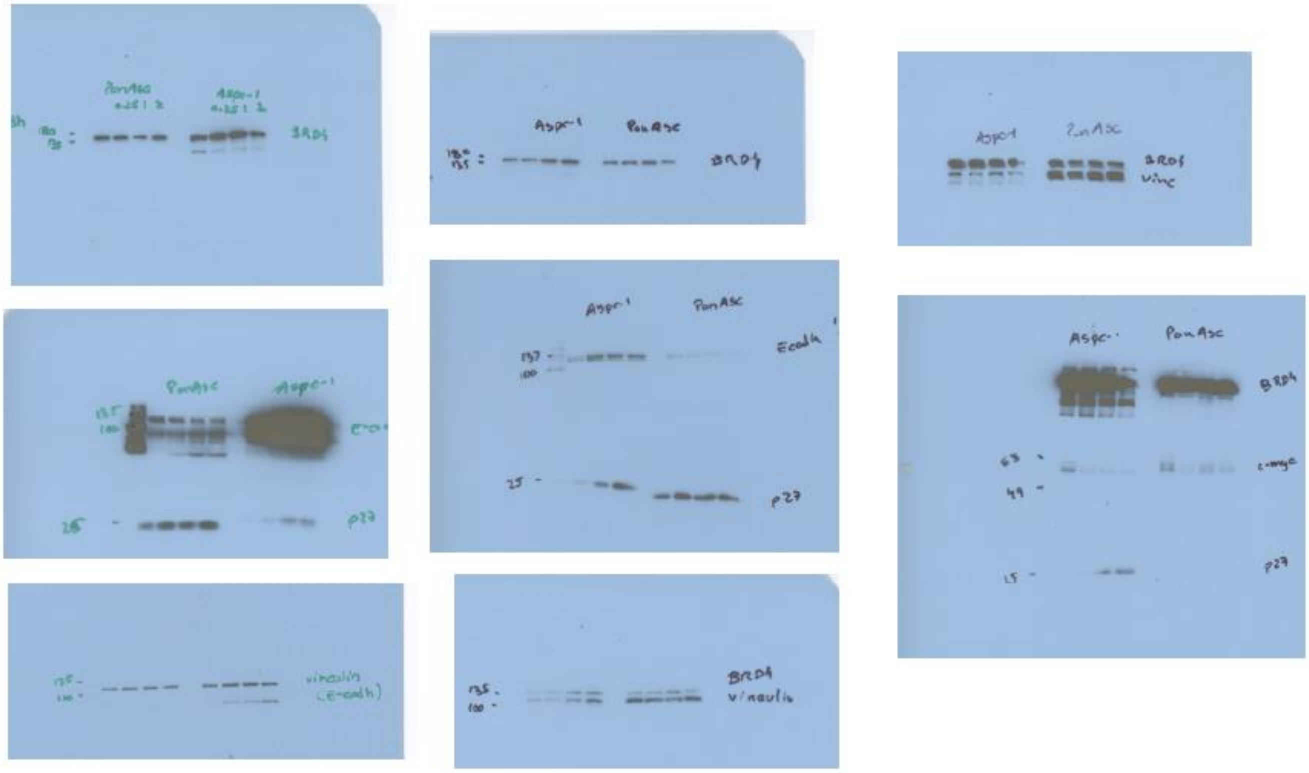

Figure S5. Uncropped Western Blots for PanAsc 2159 and Aspc-1 experiments, Figure 1 and Figure S1.

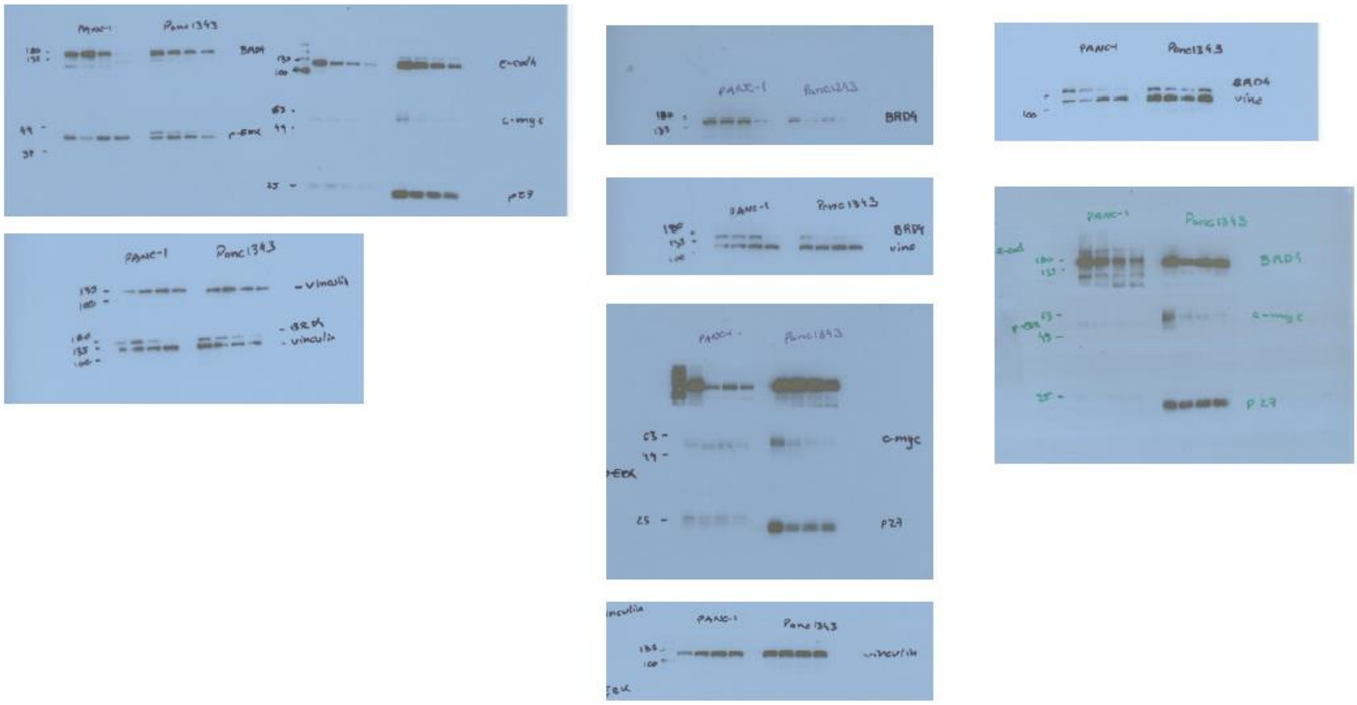

Figure S6. Uncropped Western Blots for PANC-1 and Panc 1343 experiments, Figure 1 and Figure S1.

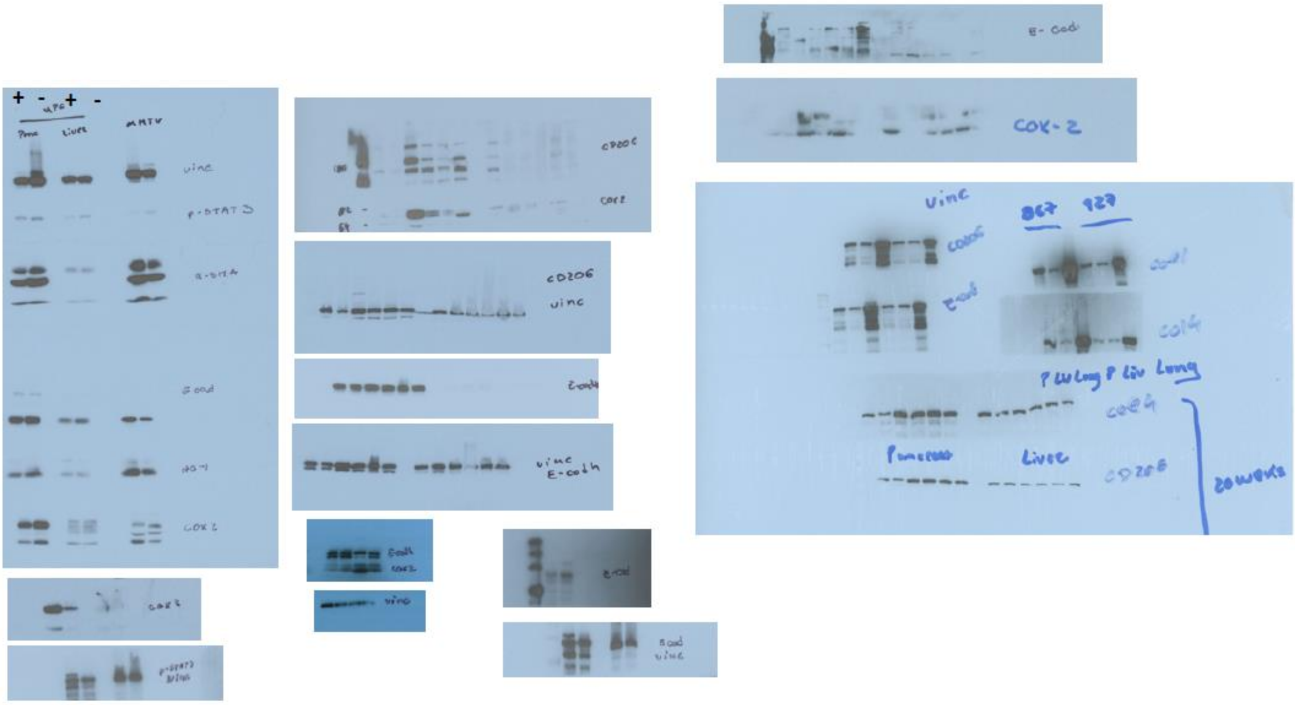

Figure S7. Uncropped Western Blots for pancreas extracts for Figure S2.

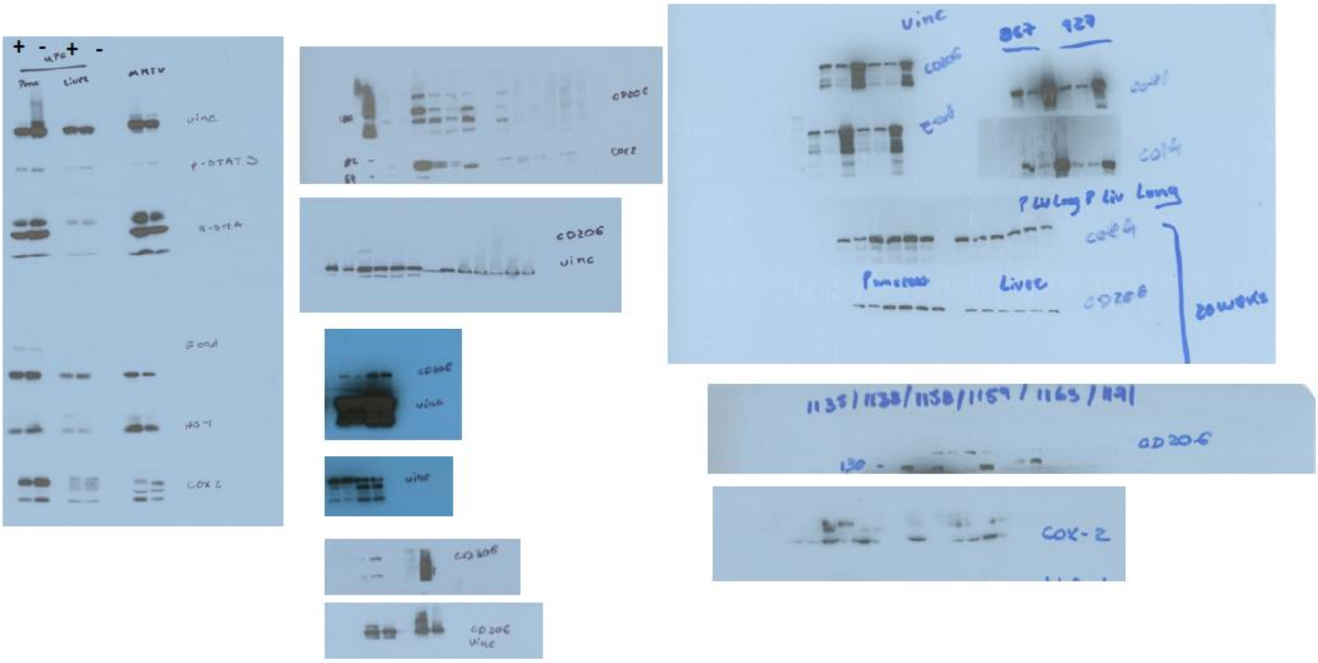

Figure S8. Uncropped Western Blots for Liver for Figure S2.

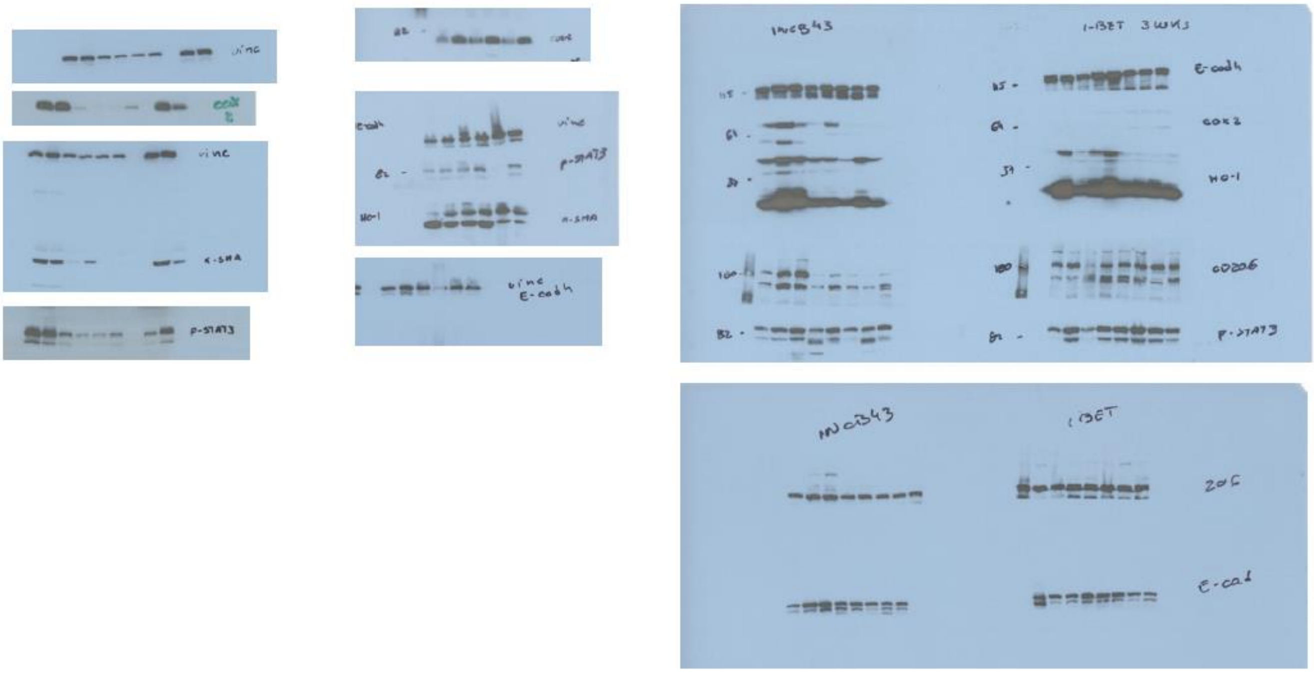

Figure S9. Uncropped Western Blots for Figure 4.

Copyright: © 2020 by the authors. Licensee MDPI, Basel, Switzerland. This article is an open access article distributed under the terms and conditions of the Creative Commons Attribution (CC BY) license (http://creativecommons.org/licenses/by/4.0/).
